# Chloride Transport Behaviour and Service Performance of Cracked Concrete Linings in Coastal Subway Tunnels

**DOI:** 10.3390/ma14216663

**Published:** 2021-11-04

**Authors:** Sulei Zhang, Qing Xu, Rui Ren, Jiahao Sui, Chang Liu, Changfeng Yuan

**Affiliations:** 1 School of Civil Engineering, Qingdao University of Technology, Qingdao 266033, China; wangyantumu@qut.edu.cn (Q.X.); dumingqing@qut.edu.cn (J.S.); yuanchangfeng@qut.edu.cn (C.Y.); 2 School of Highway, Chang’an University, Chang’an 710061, China; 3 Qingjian Goup Co., Ltd., Qingdao 266071, China; 4 Key Laboratory for Urban Underground Engineering of Ministry of Education, Beijing Jiaotong University, Beijing 100044, China; 19115030@bjtu.edu.cn

**Keywords:** subway tunnel, chloride penetration, indoor experiment, numerical analysis, service life prediction, treatment measures

## Abstract

The concrete lining in subway tunnels often undergoes cracking damage in coastal cities. The combination of cracked tunnel lining structures and high concentrations of corrosive ions in the groundwater (e.g., chlorine) can accelerate concrete erosion, reduce the mechanical performance of the lining structures and shorten the tunnel service life. This paper investigates the chloride ion concentration in the groundwater of several subway tunnels in the coastal city of Qingdao, China. Indoor experiments and numerical simulations are conducted to investigate the chloride ion transport behaviour and service performance of cracked concrete linings. The results are applied to predict the service life of lining structures. The crack depth in concrete linings is found to have the most significant effect on the transport rate of chloride ions, followed by the crack width. The numerical simulations are carried out using COMSOL software to study the chloride transport behaviour in cracked specimens and predict the service lifetimes of lining structures of different thicknesses, and the results correspond well with the experimental data. The durability of a concrete lining can be enhanced by increasing the thickness of the protective concrete layer. Additional measures are proposed for treating cracked concrete linings to resist chloride ion attack in subway tunnels.

## 1. Introduction

Ongoing urbanisation has increased the demand for subway tunnel construction worldwide, and numerous new tunnel systems have become operational in recent years. However, tunnels are often damaged by construction and/or environmental factors [1,2,3,4,5]. Improper design or poor construction quality may cause cracking of the tunnel lining structure and/or water leakage [6,7,8], and environmental erosion can lead to concrete carbonation, reinforcement corrosion and other structural damages, such as falling blocks. While the structural defects caused by poor design and construction reduce the tunnel durability, environmental erosion directly threatens the tunnel’s overall structural safety.

Subway tunnels located in coastal cities are particularly affected by the subsurface groundwater, which typically contains high concentrations of chloride and sulphate ions. The diffusion of free chloride ions can erode the reinforcing steel protective layer from the concrete surface to its interior, which corrodes the reinforcing steel when the chloride concentration reaches a certain threshold [9,10,11]. Subway tunnels in China are affected by the mutual coupling of soil/water loads and environmental erosion during their full life cycle (100 years), which drastically reduces their physical and mechanical indexes and leads to tunnel damage [12]. Lining cracks are a typical form of structural damage that can accelerate the chloride ion penetration rate and significantly reduce the service life of tunnel lining structures [13,14]. Cracked concrete linings and chloride ion attack processes should therefore be carefully considered when assessing a tunnel’s long-term safety.

Chloride chemically attacks concrete and produces Friedel salts onto which the remaining chloride ions directly adsorb [15]. The presence of structural cracks has a noticeable influence on chloride penetration, which accelerates the corrosion of the steel bars [16,17,18,19,20]. Relevant indoor experiments and numerical simulations have been conducted to investigate the structural deterioration of coastal city tunnels, and various beneficial conclusions have been obtained. Otieno et al. [21] showed that cracks increase the permeability of concrete specimens and accordingly accelerate chloride-induced corrosion and that the chloride content increases with increasing crack width. Lai et al. [22] performed wet–dry cycle experiments to explore the influence of crack width on chloride penetration and reported that the chloride concentration and penetration of a pre-cracked concrete beam increases with crack width. Yu et al. [13] proposed the XFEM to simulate corrosion-induced crack paths, used accelerated corrosion tests for comparative analysis and showed that the deflection angle of a transverse crack strongly affects the width and length of corrosion-induced cracks. Audenaert et al. [23,24] studied the effect of crack width and depth on chloride penetration using indoor experiments and numerical analysis and showed that notched specimens are more severely eroded by chloride than uncracked specimens and that the notch depth has a pronounced influence. Marcos-Meson [25] discussed the durability of cracked steel-fibre-reinforced concrete and demonstrated a high corrosion probability when cracks are wider than 0.5 mm. A series of studies have predicted the durability of cracked concrete (i.e., service life) in chloride-rich environments. It is also crucial to take into account the time parameter of the extent of chloride erosion. Known [26] predicted the service life of cracked reinforced concrete structures exposed to chloride penetration conditions and showed that the service life predicted using Monte Carlo probabilistic simulations is shorter than that obtained using the deterministic method. Audenaert [23] demonstrated that the presence of cracks significantly reduces the service life of concrete structures.

Previous studies have mainly focused on a single level of indoor accelerated tests or numerical simulation analysis but have neglected to compare the results obtained using a range of different methods. Furthermore, the existing literature has mainly focused on the effect of crack width on chloride ion attack conditions, while generally ignoring the impact of crack depth. Adopting a more comprehensive approach, this study determined the chloride content of groundwater in a tunnel service environment and the erosion mechanism of concrete lining cracking. Indoor experiments were performed to investigate and analyse the chloride penetration of lining structures with various crack widths and depths, and numerical simulations were conducted to verify the accuracy of the experimental results. Herein, the results are applied to predict the service life of concrete lining with different crack depths, and treatment measures are proposed to manage and maintain subway tunnels under corrosive environmental conditions.

## 2. Indoor Experiments of Chloride Penetration in Cracked Concrete Lining

### 2.1. Experimental Background

#### 2.1.1. Investigation of Chloride Ion Concentration in a Subway Service Environment

**Figure 1:**
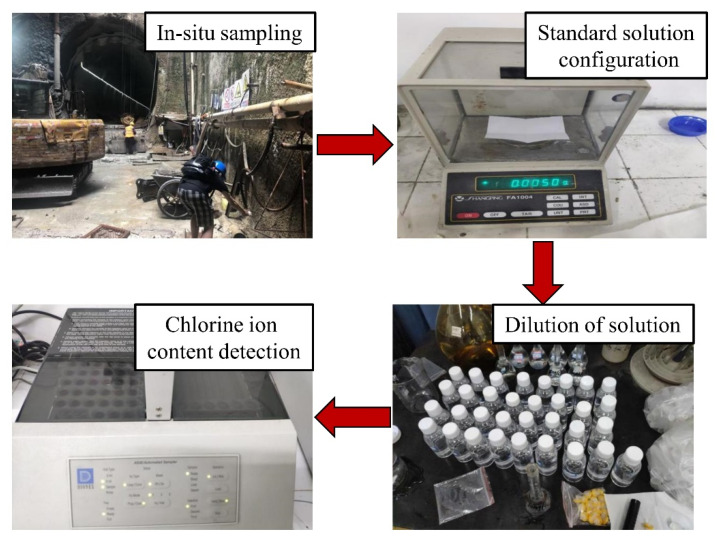
Determination of chloride ion content in subway service environments.

Numerous subway tunnels have been built throughout China, including in Qingdao City, which is located northwest of the Yellow Sea and covers an area of 11,282 km^2^. Qingdao is a coastal city, so wind and fog can transfer large amounts of corrosive ions in seawater (e.g., Cl^−^) to subway tunnels. Erosive ions are also present in the groundwater of coastal cities, all of which accelerate subway tunnel corrosion. Furthermore, chlorides can also penetrate through the concrete cover to induce the corrosion of reinforcing steel and thus deteriorate the reinforced concrete structures. Several Qingdao subway tunnels were selected as the sample to analyse the chloride content in the groundwater. Three groundwater samples were taken from each site location and maintained in clean 100 mL bottles. Further, an ICS-1500 ion chromatograph was used to determine the chloride ion content in groundwater. Firstly, the standard solutions of 5 mg/L, 10 mg/L, 20 mg/L and 50 mg/L were configured. Then, the sample solution was diluted to determine the chloride ion content (Figure 1) as

*ρ* = *A*·*D* (1)

where *ρ* is the chloride ion mass content in the water samples (mg/L), *A* is the chloride ion content in the water sample on the standard chloride ion curve and *D* is the multiple of dilution.

The chloride ion concentration in groundwater was measured in following the Code for Investigation of Geotechnical Engineering (GB 50021-2009). Among them, the chloride ion on the concrete in the steel corrosion level was divided into four levels. The specific division is presented in Table 1.

**Figure 2:**
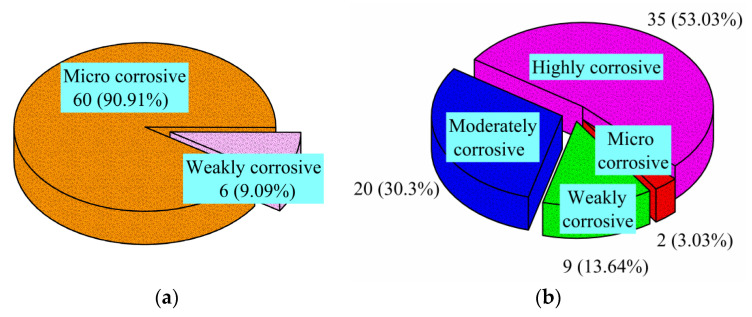
Distribution of the chloride ion content in the groundwater of subway tunnels from Qingdao: (**a**) long-term immersion; (**b**) dry–wet alternation.

Figure 2 shows the distribution of the ion detection results of 66 selected water samples from Qingdao subway tunnels. Considering long-term immersion, the corrosion of water samples could be classified as micro-corrosion and weak corrosion. The results show that 60 (90.9%) samples could be classified as micro-corrosive, whereas the remaining six samples could be classified as weakly corrosive. The corrosion classification determined from alternating wet and dry cycles could be divided into micro-corrosion, weak corrosion, moderate corrosion and high corrosion. Most of the samples could be classified as moderately corrosive and highly corrosive, accounting for 30.3% and 53.03%, respectively. Thus, some areas of the metro tunnels in Qingdao are exposed to high levels of chloride ions, which can reduce the service life of the concrete lining.

#### 2.1.2. Erosion Mechanism of Cracking Concrete Lining

**Figure 3:**
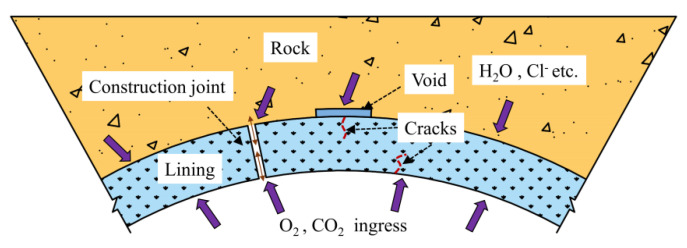
Typical working environment of a coastal tunnel.

Crack damage and water leakage can occur in subway tunnels over long operation periods. Corrosive ions in groundwater infiltrate along construction joints in the surrounding rock to the interior of the lining cracks. Air can also enter the exterior lining [23] and form a connective channel for ion corrosion. Chloride ion erosion can consequentially affect both sides of the lining cracking area (Figure 3). The chloride corrosion environment of subway tunnels was therefore taken as the research background for the indoor tests to simulate the effects of variable crack width and depth on the transport behaviour of chloride ions in the concrete lining [27,28].

### 2.2. Experimental Preparation

#### 2.2.1. Raw Materials and Mix Proportions

As the basis for determining the corrosion of a reinforcement, one can consider when the accumulated chloride ion concentration at the protective layer reaches the critical concentration [24]. The experimental samples were composed of raw materials that are characteristic of the Qingdao subway tunnels, such as cement, aggregate, fly ash and slag. Portland Cement P.O. 42.5 produced by Shandong Shanshui Cement Co. Ltd. (Qingdao, Shandong, China) was used. Continuous grading levels of gravel included coarse aggregates with a diameter of 5–25 mm, and fine aggregates of medium-sized river sand with a fineness modulus of 2.7. Polycarboxylate superplasticiser (S.P.) was used for reducing the water content. The mix proportions of the lining concrete are presented in Table 2.

#### 2.2.2. Sample Preparation

**Figure 4:**
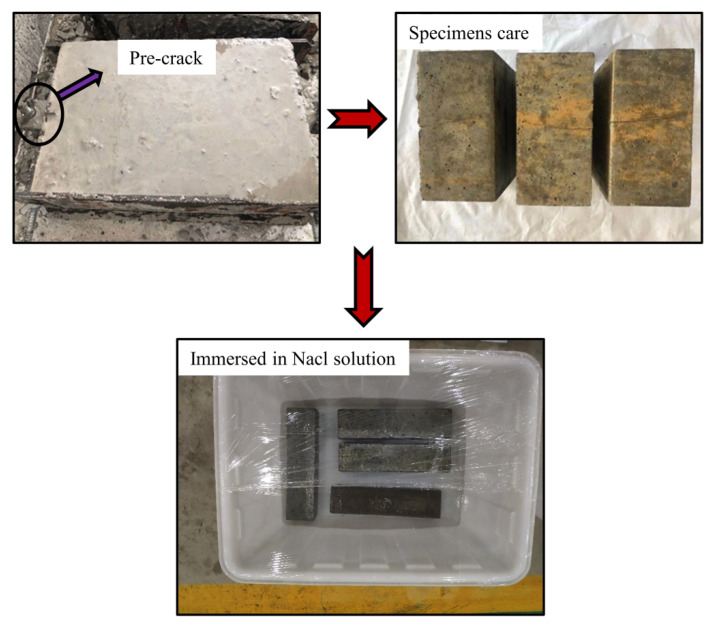
Experimental samples.

Six types of concrete specimens with dimensions of 100 mm × 200 mm × 300 mm were prepared. Table 3 lists the combinations of crack widths and depths; note that no cracks were present in the sample listed as having a crack width and depth of zero. Previous studies have shown that artificial cracks are easier to handle than natural cracks and that they can be used to draw conclusions regarding natural cracks [29]. The concrete specimens with artificial cracks were prepared using the scoring method. A thin steel sheet was positioned inside the specimen and removed approximately 4 h after pouring. The steel sheets had a thickness of 0.05 mm, 0.1 mm or 0.2 mm, and their placement in the concrete created crack depths of 5 mm, 10 mm and 20 mm, respectively. The samples were removed from the mould after 24 h and cured at constant temperature (20 ± 3 °C) and relative humidity exceeding 95% for 28 days. After 28 days of natural curing and crack production, the five surfaces of the specimens (except for the cracked surface) were sealed with epoxy to prevent the ingress of chloride ions. In a real service environment, it takes years or even decades for subway tunnels to suffer corrosion. Therefore, higher NaCl solutions than the Cl^−^ concentration in real groundwater are necessary to accelerate the process under indoor experimental conditions. The concrete specimens were immersed in a corrosive solution of 5% NaCl for 90 days (Figure 4). In addition, the solution was renewed every 15 d to ensure that the concentration and pH in the solution were stable. The average temperature was 16 °C and relative humidity was approximately 79% for the indoor environment.

**Figure 5:**
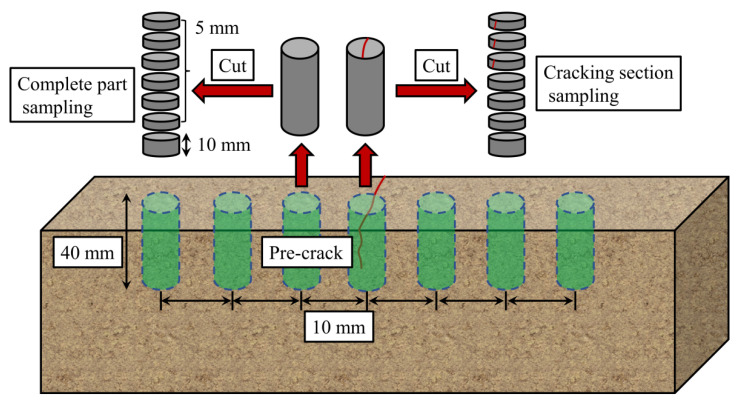
Concrete specimen sampling.

After 90 days, the specimens were removed from the solution and powdered in layers. The cracked section was sampled along its two sides at distances of 10 mm, 20 mm and 30 mm to analyse the experimentally induced chloride penetration profiles. The exposed surface was also analysed for the sample core by grinding the powder in 5 mm increments until reaching 30–40 mm depth (Figure 5). These analytical steps were performed in accordance with the Test Code of Standard for Long-Term Performance and Durability Test Method of Ordinary Concrete (GB/T 50082-2009).

### 2.3. Results and Discussion

#### 2.3.1. Effect of Crack Width and Length on Chloride Distribution

**Figure 6:**
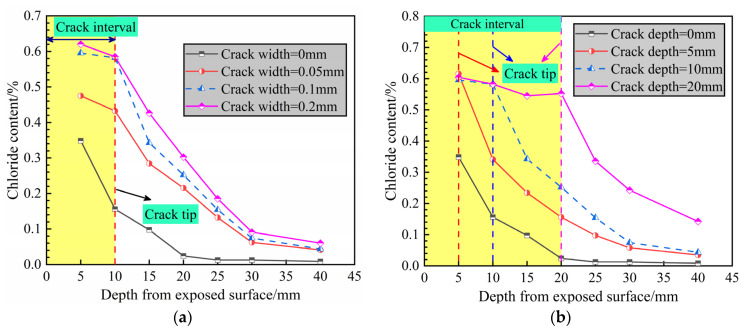
Chloride content depth profiles for concrete specimens with different (**a**) crack widths and (**b**) crack lengths.

The chloride migration experiments were terminated after 90 days. The chloride concentration profiles in the middle of specimens (i.e., cracked sections) are shown in Figure 6 for a series of crack widths (Figure 6a) and depths (Figure 6b). The chloride content in concrete is based on the percentage of cement weight. The chloride content decreases with increasing depth. Figure 6a shows the chloride profiles for crack widths of 0 mm (i.e., sound concrete), 0.05 mm, 0.1 mm and 0.2 mm at a fixed crack depth of 10 mm. The chloride content was considerably high near the exposed surface and slightly increased with increasing crack width. The chloride penetration rate in the cracked specimens was greater than that of the intact sample because the cracks provided a fast pathway for ion transport. Upon reaching the concrete protective layer thickness (40-mm depth), the chloride ion concentration was approximately 0.06% for a crack width of 0.2 mm, slightly higher than that of the sound concrete.

Figure 6b shows the chloride concentration profiles for specimens with crack depths of 0 mm (i.e., sound concrete), 5 mm, 10 mm and 20 mm at a fixed crack width of 0.1 mm. The free chloride ions in the solution quickly eroded into the crack tip, resulting in high permeability within the crack interval. Similarly, higher chloride contents were noted for specimens with greater crack depths. Upon penetrating to 40 mm, the chloride content of the specimen with a crack depth of 20 mm reached 0.14%, which is substantially greater than that of the intact specimen. This demonstrates that crack depth has a more pronounced effect on chloride penetration than crack width, which is consistent with the findings of [23,24].

**Figure 7:**
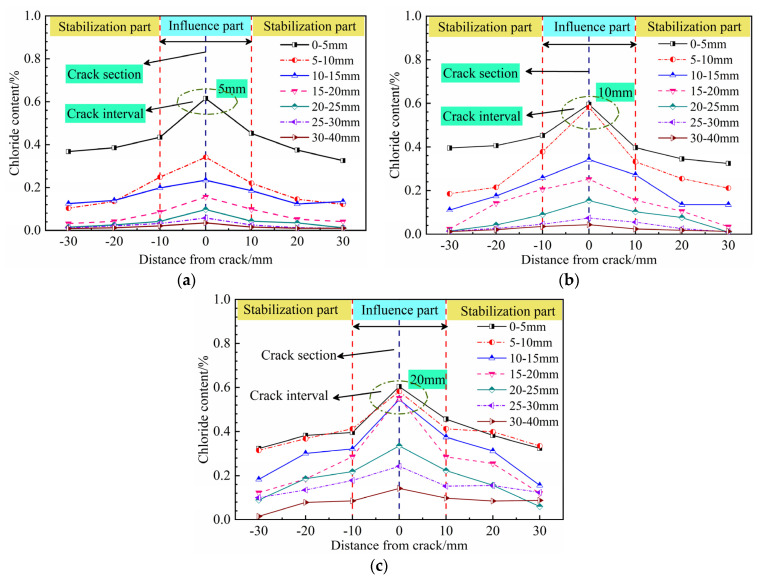
Two-dimensional chloride concentration profiles for specimens with crack depths of (**a**) 5 mm, (**b**) 10 mm and (**c**) 20 mm.

Figure 7 illustrates the two-dimensional chloride concentration profiles for concrete specimens with different crack depths. The variation in chloride ion concentration generally exhibited a normal distribution, in which the chloride concentration decreased with increasing distance from the crack. The chloride content varied sharply within the distance interval of −10 mm to 10 mm from the crack and was stable at distances greater than 10 mm from the crack. In the crack cross-section, the chloride content was significantly larger in the cracked interval than in other locations of the horizontal section, such as the point at 0–5 mm for the specimen with a crack depth of 5 mm (Figure 7a) and the four points between 0 and 20 mm for the specimen with a crack depth of 20 mm (Figure 7c). The cracked concrete clearly presented a two-dimensional diffusion profile, showing that chloride ion erosion occurred most severely in the cracked section. The subsequent analyses were therefore focused on chloride penetration in the crack cross-section.

#### 2.3.2. Chloride Diffusion Coefficient in Cracked Specimens

The chloride diffusion rate in sound concrete is confirmed following Fick’s second law [30], and the total chloride content can be expressed as

(2) $$C_{x,t}=C_{0}+(C_{sa}-C_{0})\left[1-erf\left(\frac{x}{2\sqrt{Dt}}\right)\right]$$

where $C_{x,t}$ is the chloride content at depth *x* and exposure time *t*, $C_{0}$ is the initial chloride content, $C_{sa}$ is the surface chloride content and *D* is the chloride diffusion coefficient.

The propagation of chloride ions in concrete is also affected by cracks. In such cases, the chloride diffusion coefficient *D* can be replaced by *D*(*w*), and the correlations between the equivalent chloride diffusion coefficient and deterioration factor *f*(*w*) for specimens with cracks can be described as [31,32]

(3) $$D\left(w\right)=f\left(w\right)\times D_{0}$$

where *D*(*w*) is the chloride diffusion of cracked specimens, *D*_0_ is the chloride diffusion of intact specimens and *f*(*w*) is the deterioration factor.

The calculated values are listed in Table 4. The fast transport passage provided by the cracks clearly accelerates the chloride erosion rate, and the chloride diffusion coefficient in the cracked specimens is greater than that of the intact specimens. For a fixed crack depth of 10 mm, *D*(*w*) increases with increasing crack width and reaches 23.2607 × 10^−12^ m^2^/s for a crack width of up to 0.2 mm, which is 3.88 times higher than that of the intact concrete. For a fixed crack width of 0.1 mm, the *D*(*w*) values increase with crack depth, reaching 28.0135 × 10^−12^ m^2^/s for the specimen with a crack depth of 20 mm, for which the deterioration factor *f*(*w*) is 4.67. Crack depth is therefore found to have a more pronounced effect on the *D*(*w*) values than crack width.

## 3. Numerical Simulations

### 3.1. Model Establishment

**Figure 8:**
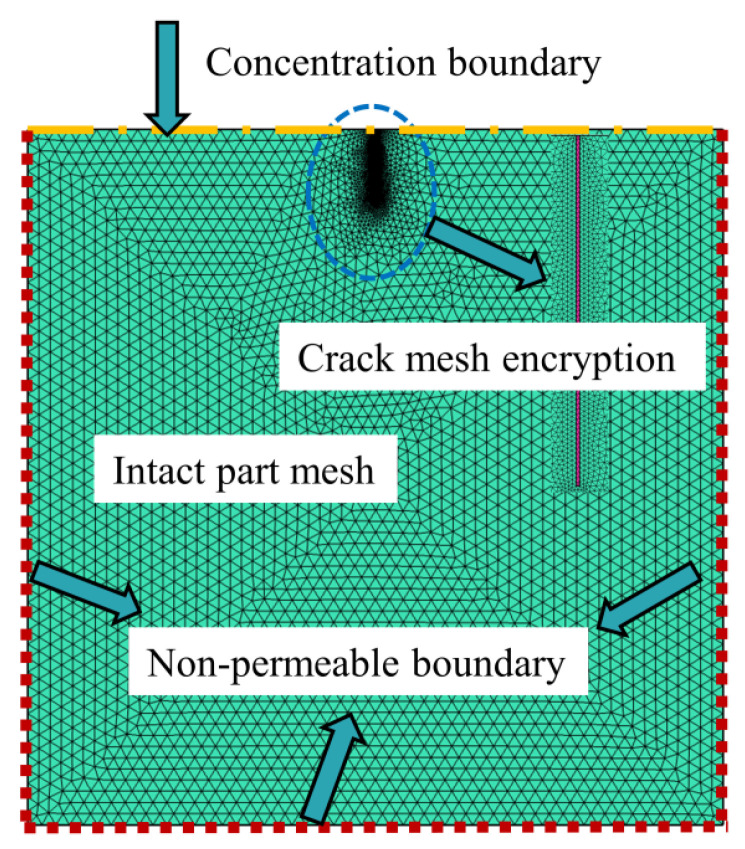
Mesh divisions.

The numerical simulations to calculate the chloride content of concrete specimens were performed on finite element software COMSOL. In the simulations, the actual crack geometry was simulated and the mesh was encrypted (Figure 8). The aim of the simulations was not only to compare and verify the experimental data but also to explore the service life of the cracked concrete specimens. The chloride diffusion model and parameter settings were formulated as follows.

(1) Concrete rarely contains appreciable chloride ions in the initial conditions; thus, the initial chloride content inside the specimen was set to *C*(*x*,0) = 0.
(2) The boundary condition of the surface chloride content was set to be consistent with the indoor experiments.
(3) Chloride penetration represents the ability of free chloride ions to diffuse from high to low concentrations in the specimen. The chloride diffusion coefficient is greater in the cracked areas than in the uncracked areas. These areas are thus defined separately based on the experimental data.
(4) Transient analysis was used because the chloride content in the specimens varied with time. The transient analysis of each time step was chosen as one day for a total time of 90 days.

### 3.2. Results and Discussion

**Figure 9:**
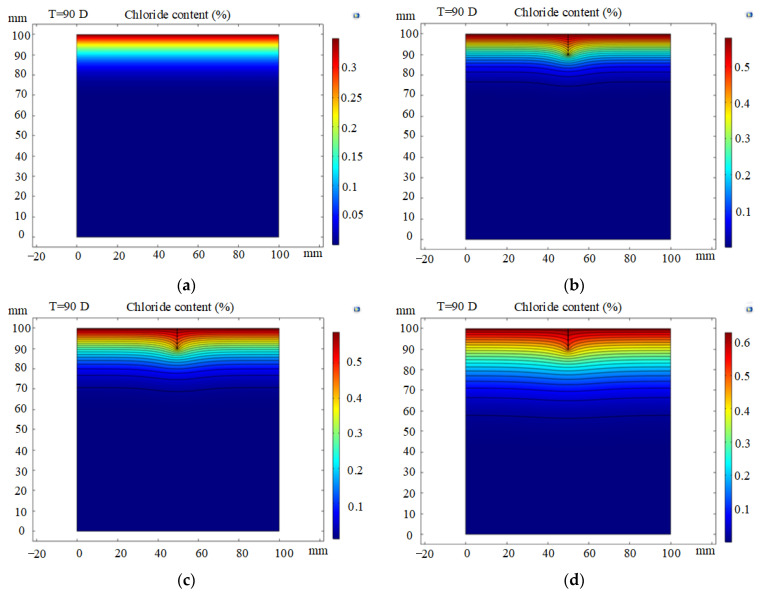

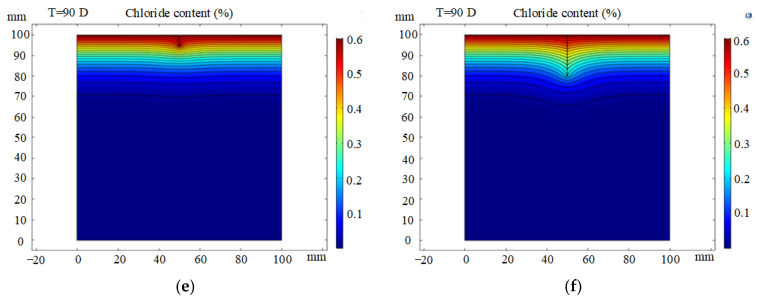
Simulated chloride distribution contours in cracked concrete lining: (**a**) crack width = 0 mm, crack depth = 0 mm (i.e., sound concrete); (**b**) crack width = 0.05 mm, crack depth = 10 mm; (**c**) crack width = 0.1 mm, crack depth = 10 mm; (**d**) crack width = 0.2 mm, crack depth = 10 mm; (**e**) crack width = 0.1 mm, crack depth = 5 mm; (**f**) crack width = 0.1 mm, crack depth = 20 mm.

Figure 9 shows the two-dimensional concentration contours for six simulations with different crack widths and depths. In the intact concrete simulation, the diffusion form of the chloride ion is one-dimensional, and its concentration decreases with increasing depth from the exposed surface, as shown in Figure 9a. In contrast, higher chloride contents are obtained at the crack tip than in the intact section. The chloride content in the cracked concrete shows a similar normal distribution curve with high concentrations in the middle and low concentrations in the ends. In Figure 9b–f, the contours tend to flatten with increasing depth from the exposed surface. Chloride erosion in the cracked area therefore has an apparent two-dimensional diffusion characteristic, as described in Section 2.3.1.

Figure 9b,d show the chloride concentration distribution at a fixed crack depth of 10 mm and crack widths of 0.05 mm, 0.1 mm and 0.2 mm, respectively. Greater chloride ion penetration is associated with larger crack widths. Similarly, the penetration depth increases with increasing crack depth. This is because chloride ions can quickly enter the lining along the ‘fast track’, as shown in Figure 9e,f. Crack depth is found to have a more pronounced effect on chloride penetration than crack width, which is consistent with the experimental results. Crack depth is therefore a major influential factor for the service life of concrete.

**Figure 10:**
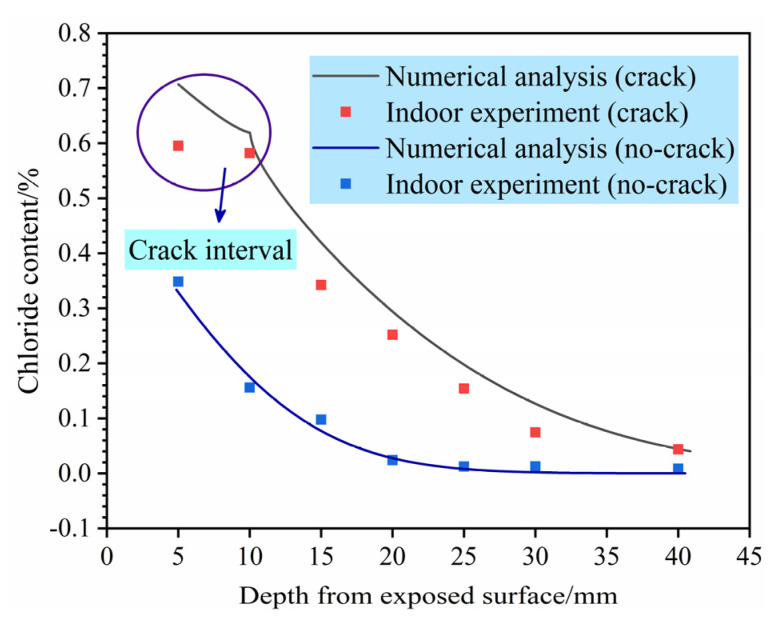
Comparison of chloride content determined analytically and experimentally.

Figure 10 compares the experimental and simulation results for sound concrete and concrete with a 10-mm-deep and 0.1-mm-wide crack. The agreement is reasonable considering the simulation assumptions and experimental unknowns and variability. Specimens with a crack width of 0.1 mm and crack depths of 0 mm, 5 mm, 10 mm and 20 mm were analysed for predicting the service life of concrete.

### 3.3. Prediction of Service Life of Cracked Concrete Lining

#### 3.3.1. Prediction Guidelines

Previous studies have shown that when the chloride ion content reaches a critical value within the protective layer steel bar, the passivation layer is damaged and chloride ion corrosion occurs on the steel bar surface [13]. In this study, the critical chloride ion concentration was converted to 0.15% of the weight of cement [33], and the chloride surface concentration was set to 0.45%. It is also crucial to take into account the time parameter of the extent of chloride erosion. The diffusion coefficient of chloride ions in free solution is 1.8 × 10^−9^ m^2^/s. Alternatively, the diffusion coefficient in the cracked part can be considered equal to that in free water [34,35]. The foregoing analysis demonstrates that crack depth is the main factor that affects the chloride concentration; thus, the crack width was set to 0.1 mm and crack depths of 5 mm, 10 mm and 20 mm were selected for calculation and comparison with an intact lining structure. A transient solver was used to calculate the chloride ion diffusion process over a 100-year period.

#### 3.3.2. Analysis of Service Life Prediction Results

The Code for Metro Design (GB50157) stipulates that the protective layer thickness of a tunnel lining should not be less than 40 mm. However, for more conservative considerations, subway lining structure protection layer thicknesses are often set to at least 50 mm. Protection layer thicknesses of 40 mm and 50 mm were therefore analysed.

**Figure 11:**
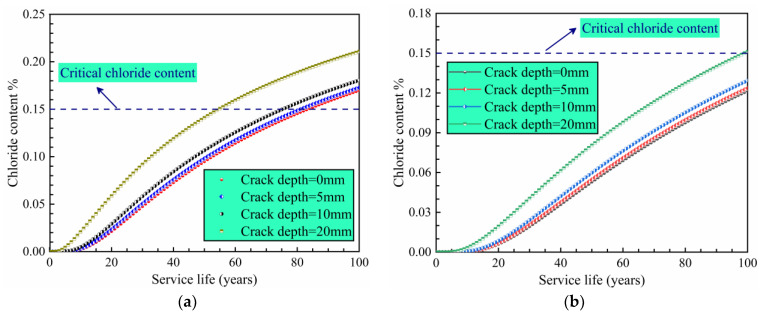
Chloride content as a function of time in a structure protection layer with a thickness of (**a**) 40 mm and (**b**) 50 mm.

Figure 11 shows the chloride content at different protective layer thicknesses as a function of time for crack depths from 0 to 20 mm. Figure 11a shows that 84 years are required for chloride to reach the critical content value in intact concrete. For a crack depth of 20 mm, the chlorine corrosion process starts after only 55 years; the service life is thus reduced by 34% compared with the intact concrete. The results indicate that a 40-mm-thick protective layer does not satisfy the service life requirement of 100 years. When the protection layer thickness is set to 50 mm (Figure 11b), the samples with crack depths of 0 mm, 5 mm and 10 mm do not reach the critical chloride content (0.15%). In contrast, for a crack depth of 20 mm, the concentrations are slightly higher than 0.15%, thereby failing to meet the service life requirements. These results further support the notion that crack depth has a significant influence on the service life of the lining structure.

**Figure 12:**
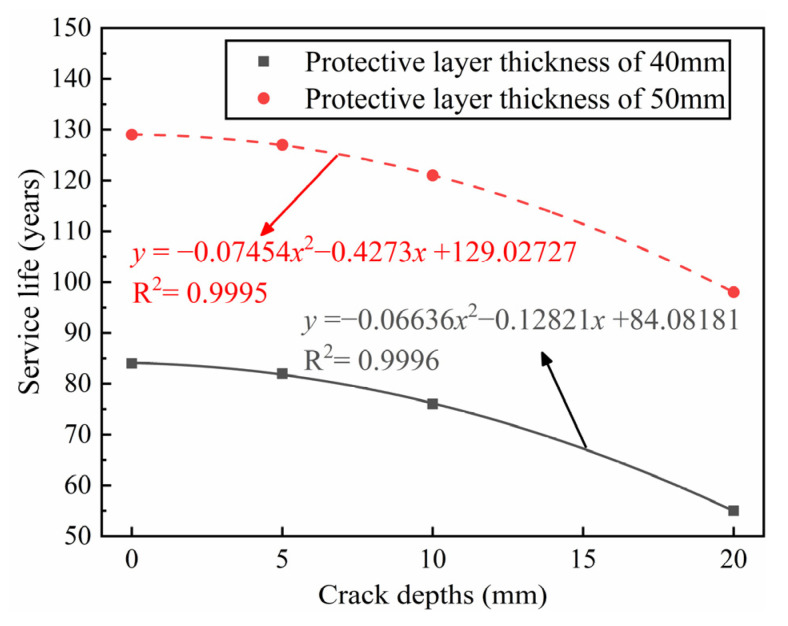
Service life of structures with a different protective layer thickness and crack depth.

Figure 12 describes the relationship between crack depth, protective layer thickness and service life. A thicker protective layer drastically improves the service life of the structure, and deeper cracks are shown to reduce the service life. The design thickness of the protection layer in the Qingdao metro is 50 mm, which meets the service life requirement when the crack depth is less than 20 mm. However, if the crack depth exceeds this value, it is not realistic to meet the service life requirements by increasing the protective layer thickness, and other treatment measures are therefore required.

## 4. Treatment Measures for Structural Durability

The foregoing analysis clearly indicates that cracks in chloride corrosion environments directly affect the service life of a tunnel structure. Several treatment measures have therefore been designed to fulfil the structural durability requirements.

### 4.1. Voids behind the Lining

**Figure 13:**
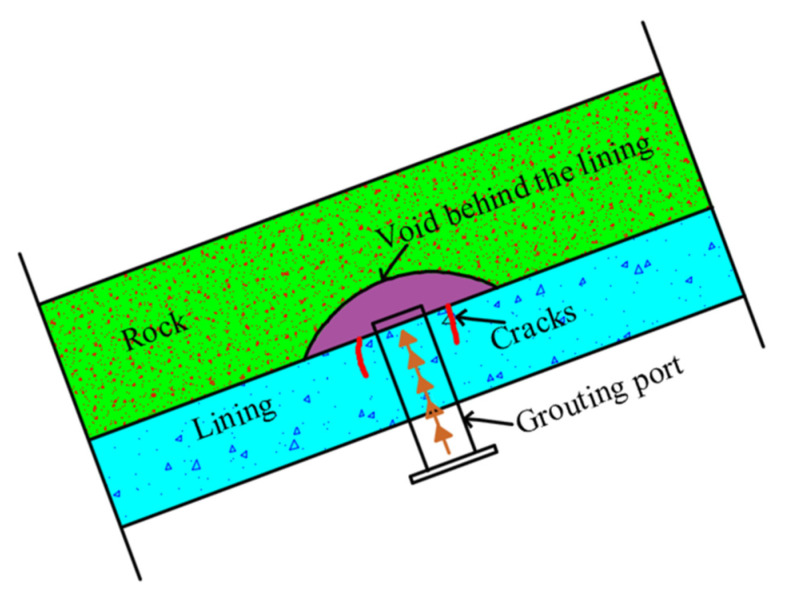
Grouting of a void behind the lining.

During the operation of a subway tunnel, loose areas of the surrounding rock may fall and form a void behind the lining owing to subway vehicle vibration and/or groundwater scouring effects. This can lead to lining cracks, water leakage and other structural diseases. To address this problem, it is recommended to backfill the void with grout (Figure 13). Specifically, grouting holes can be drilled at lengths that can be adjusted according to the onsite void distribution. To ensure that the voids are densely filled, it is necessary to inject grouting materials, such as traditional double slurry, chemical slurry and foam concrete, through the grouting holes.

### 4.2. Lining Surface Cracks

**Figure 14:**
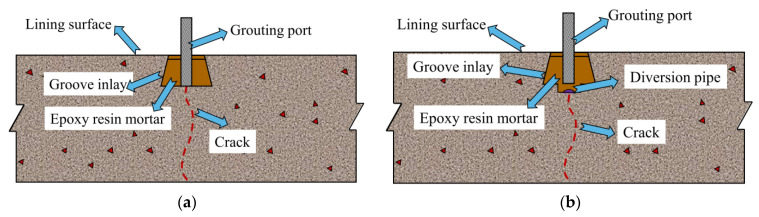
Crack treatment on lining surface: (**a**) no seepage; (**b**) seepage.

For cracks on the surface of a subway lining, a wedge-shaped groove can be used in the cracked area to cut the groove inlay treatment. Epoxy resin mortar can be injected into the cracks by applying a certain grouting pressure to the grouting pipe to fill the crack gap, as shown in Figure 14a. In the case of water seepage in the cracked area, the cracks in the seepage area can be treated by excavation and hydraulic diversion-slurry filling to avoid lining structure erosion owing to the high concentration of chloride ions in groundwater. As shown in Figure 14b, the grooved area is connected to the permeable water pipe, and drainage is realised through the diversion pipe, followed by slurry sealing.

## 5. Conclusions

This study investigated the service environment of coastal subway tunnels and the chloride ion concentration in the groundwater. Indoor experiments were carried out to determine the impact of lining crack width and depth on the chloride transport behaviour in the concrete lining. In this paper, the experimentally induced chloride compositional profiles are compared with the numerical simulation results, yielding a reasonable agreement. The service life of a concrete lining in a chloride-rich environment is predicted for a range of crack width and lining thickness conditions. The main conclusions are summarised as follows.

(1) An inspection of subway tunnels in coastal cities indicates that these service environments often contain high concentrations of chloride ions.
(2) Indoor experiments show that the chloride ion concentration in a concrete protective layer increases with increasing crack width and crack depth. The effect of crack depth on chloride ion attack in the protective layer is therefore more substantial than that of crack width.
(3) According to numerical simulations performed to calculate the chloride permeability of a cracked lining structure and to predict its service life, the service life of an intact concrete protection layer with a thickness of 50 mm is 129 years, which meets the structural durability requirements. However, for crack depths that exceed 20 mm, the service life is less than 100 years. Crack depth therefore has a significant impact on the service life of subway tunnels.
(4) Measures to effectively treat subway tunnel damage can enhance the tunnel safety and service life. A series of treatment measures are proposed herein to improve the durability of the lining structure caused by tunnel damage.

## Figures and Tables

**Table 1:** Evaluation of chloride ion concentration on corrosion of steel reinforcement.

Corrosion Level	Chloride Ion Concentration in Groundwater (mg/L)	
	Long-Term Immersion	Alternating Wet and Dry Cycles
Micro-corrosion	<10,000	<100
Weak corrosion	10,000–20,000	100–500
Moderate corrosion	-	500–5000
High corrosion	-	>5000

**Table 2:** Mix proportions of lining concrete (kg/m^3^).

NO.	Cement	*w/b*	Fine Aggregate	Coarse Aggregate	Water	S.P.	F.A.	S.L.
C45	360	0.35	750	1035	158	6.3	40	50

**Table 3:** Pre-crack sizes.

Table.	Crack Width (mm)	Crack Depth (mm)
1	0	0
2	0.05	10
3	0.1	10
4	0.2	10
5	0.1	5
6	0.1	20

**Table 4:** Equivalent chloride diffusion coefficients of cracked specimens.

Crack Depth (mm).	Crack Width (mm)	*D*(*w*) (×10^−12^ m^2^/s)	*f*(*w*)	*R* ^2^
0	0	6.0018	1	0.9905
5	0.1	10.8619	1.81	0.9861
10	0.05	16.3474	2.72	0.9772
10	0.1	20.1550	3.36	0.9896
10	0.2	23.2607	3.88	0.9679
20	0.1	28.0135	4.67	0.9764

## Data Availability

All data has been included in the paper.
